# C-reactive protein as a marker of persistent *Chlamydia trachomatis* infection is not associated with tubal factor infertility—an independent clinical validation study

**DOI:** 10.1093/hropen/hoz029

**Published:** 2019-12-16

**Authors:** Me Jansen, Ef van Ess, S Ouburg, Ml Gerds, Sa Morré, Ja Land

**Affiliations:** 1 Institute for Public Health Genomics, Department of Genetics and Cell Biology, School for Oncology and Developmental Biology (GROW), Faculty of Health, Medicine and Life Sciences, Maastricht University, 6229 ER, Maastricht, The Netherlands; 2 Amsterdam UMC, Location VUmc, Department of Medical Microbiology and Infection Control, Laboratory of Immunogenetics,1081 HZ, Amsterdam, The Netherlands; 3 Amsterdam UMC, Location VUmc, Department of Clinical Genetics, Section Community Genetics, Amsterdam Public Health Research Institute, 1081 HV, Amsterdam, The Netherlands; 4 Department of Emergency Medicine, Hospital Tjongerschans, 8441 PW Heerenveen, The Netherlands

**Keywords:** tubal factor infertility, *Chlamydia trachomatis*, C-reactive protein, *Chlamydia trachomatis* antibody test, fertility workup, low-grade inflammation, screening

## Abstract

**STUDY QUESTION:**

Does C-reactive protein (CRP), as a marker of persisting low-grade inflammation, identify *Chlamydia trachomatis* IgG antibody test (CAT)-positive women who are at the highest risk for tubal factor infertility (TFI)?

**SUMMARY ANSWER:**

No association was found between slightly elevated CRP (seCRP) levels and TFI in our CAT-positive patient population.

**WHAT IS KNOWN ALREADY:**

In the fertility work-up, CAT is used to estimate the risk for TFI and to select high-risk patients for additional invasive diagnostic procedures (e.g. hysterosalpingography and laparoscopy). However, a high number of false positives exist among CAT-positive patients. In a previous study, it has been suggested that women with TFI may be identified more accurately when combining CAT with CRP, a marker for persistent low-grade inflammation.

**STUDY DESIGN, SIZE, DURATION:**

Our original retrospective cohort consisted of 887 consecutive female patients who visited the fertility clinic of a tertiary care centre between 2007 and 2015. All CAT-positive women who underwent laparoscopy (as the reference test for evaluation of tubal function) and who had not undergone previous pelvic surgery were included in the study. CRP was determined in spare serum samples, and medical data was obtained by chart review.

**PARTICIPANTS/MATERIALS, SETTING, METHODS:**

A total of 101 women (11.4%) were CAT-positive, and 64 of these 101 women (7.2%) met all inclusion criteria. CAT was performed with an ELISA. TFI was assessed by laparoscopy and strictly defined as extensive peri-adnexal adhesions and/or distal occlusion of at least one tube. In spare sera, CRP was performed with a high-sensitivity CRP ELISA, and CRP levels between 3 and 10 mg/L were defined as positive. Analyses were corrected for BMI, endometriosis and smoking.

**MAIN RESULTS AND THE ROLE OF CHANCE:**

There was no statistically significant association between seCRP level and TFI after adjusting for BMI, endometriosis and smoking (odds ratio 1.0; 95% CI 0.3–3.3; *n* = 64).

**LIMITATIONS, REASONS FOR CAUTION:**

Our retrospective study had a small sample size due to a low CAT-positivity rate and a conservative clinical policy with regard to invasive diagnostic testing. Additionally, CRP levels were only measured once, while they may change throughout the menstrual cycle and in time.

**WIDER IMPLICATIONS OF THE FINDINGS:**

Contrary to previous findings, our results show CRP is not suitable as a marker of persistent low-grade inflammation in CAT-positive women. Other inflammatory markers and immunogenetic host factors should be studied on their clinical validity and utility to improve non-invasive risk assessment for TFI in the fertility work-up.

**STUDY FUNDING/COMPETING INTEREST(S):**

This work was partially supported by the European EuroTrans-Bio Grant [Reference number 110012 ETB] and the Eurostars grant (E!9372). S.A.M., a full-time employee of Amsterdam University Medical Centres location VUMC (0.56 fte) and the Maastricht University Medical Center (0.44 fte), is the founder (2011) and CEO of TubaScan Ltd, a spin-off company, Dept. of Medical Microbiology and Infection Prevention, Amsterdam UMC, location VUmc, Amsterdam, the Netherlands. S.O. and E.F.v.E. at the time of conducting this research had a partial appointment at TubaScan Ltd.

**WHAT DOES THIS MEAN FOR PATIENTS?**

The fallopian tubes act as an internal channel for the movement of eggs and sperm. Chlamydia infection can damage the fallopian tubes, causing tubal factor infertility (TFI). TFI is best diagnosed by passing dye through the neck of the womb into the tubes to see if they are open, but such a test can be invasive, painful and expensive. A blood test (called CAT) can be abnormal in some women who are more likely to have TFI, but many women who test positive do not actually have this condition. It has been suggested that adding a second test for a protein called C-reactive protein (CRP) could help to improve its accuracy. In this study, we used the CRP test in a group of women in whom the CAT test was abnormal and who then underwent an accurate test for TFI by means of keyhole surgery (laparoscopy). Our results suggest that this extra blood test does not improve our chances of diagnosing TFI in women with an abnormal CAT test result.

## Introduction

The role of *Chlamydia trachomatis* in tubal factor infertility (TFI) is well established. *C. trachomatis* antibodies can be detected in 67–84% of women with TFI (Broeze *et al*., 2011; Keltz *et al.*, 2013), and *C. trachomatis* IgG antibody testing (CAT) was introduced in the fertility work-up to identify patients at high risk for TFI in a non-invasive way. CAT was shown to have a high negative predictive value and specificity, both reported around 80–90% (Broeze *et al.*, 2011; Land *et al.*, 2003), which makes it suitable to identify infertile women without TFI. However, the positive predictive value and sensitivity have been reported to be around 50% (Broeze *et al.*, 2011; Land *et al.*, 2003), which makes CAT less useful in identifying women who have tubal pathology. Since CAT-positive women are generally offered an invasive diagnostic procedure, i.e. hysterosalpingography (HSG) or laparoscopy, false-positive results should be minimised as they may lead to unnecessary, painful and expensive procedures.

CAT is a marker of a previous *C. trachomatis* infection, but CAT is not informative about the course of the infection (Budrys *et al.*, 2012). *C. trachomatis* IgG antibodies may be present in serum after a short, fast-cleared infection that most likely will not result in tubal damage. It may, however, also give rise to a persistent infection that may induce severe TFI (Hjelholt *et al.*, 2011). Therefore, a more accurate non-invasive screening test (combination) for TFI is needed, and a marker that indicates the course and severity of a previous *C. trachomatis* infection might improve the accuracy of predicting the risk of tubal damage and allow a better preselection of patients for additional invasive diagnostic procedures.

C-reactive protein (CRP) is excreted by hepatocytes in case of inflammation and is a marker for tissue damage (Gabay and Kushner, 1999). CRP levels are increased during acute inflammation (CRP ≥ 10 mg/L), but slightly elevated CRP (seCRP) levels (3–10 mg/L) are considered to reflect a persistent low-grade inflammation (Gabay and Kushner, 1999; Kushner and Antonelli, 2015). CRP levels have been used in cardiovascular disease to identify and monitor patients with ongoing inflammation, for example in atherosclerotic plaque formation (Pearson *et al*., 2003). In cardiovascular disease patients, seCRP was confirmed as a proxy for higher risk of stroke and cardiovascular events (Johnston *et al.*, 2001).

In infertile women, den Hartog *et al*. (2005) found CAT-positive women with seCRP levels to be at higher risk for TFI compared to CAT-positive women without elevated CRP (odds ratio (OR) 39.7, 95% CI 11.2–140.5). This promising result in a small cohort of 22 CAT-positive women has not been confirmed so far. We aim to evaluate the association between seCRP levels and TFI in CAT-positive women in an independent cohort. We restricted our study to CAT-positive women only since CAT-negative women are considered not to have had a previous *C. trachomatis* infection and a marker for persistent infection is therefore not relevant.

## Materials and Methods

### Study population

**Figure 1:**
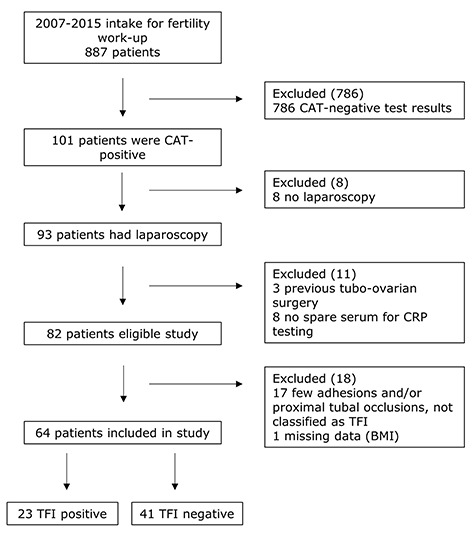
**Flow diagram showing the selection of CAT-positive patients for a study of C-reactive protein in the identification of women at high risk of TFI.** CAT: *Chlamydia trachomatis*, IgG antibody test, CRP: C-reactive protein, TFI: tubal factor infertility.

We studied a cohort of consecutive female patients who visited the fertility clinic of the University Medical Centre Groningen (UMCG) between 2007 and 2015 for a fertility work-up. Blood was drawn for CAT from all patients at their initial visit, and spare serum was cryopreserved at −20°C. Patients with a positive CAT were offered laparoscopy with methylene blue dye testing as part of their fertility work-up, unless anovulation or severe male factor infertility (requiring IVF/ICSI) was diagnosed. Only women with positive CAT results who had undergone laparoscopy as part of their fertility work-up, of whom spare serum was available for CRP testing and who had not undergone previous pelvic surgery (except for an uneventful appendectomy or Caesarean section) were included in the present study (Fig. 1). Relevant medical data were retrospectively collected from patient files including characteristics that may influence CRP levels, such as chronic diseases, endometriosis (peritoneal lesions with adhesions, endometrioma or deep infiltrating endometriosis), smoking status (cigarettes/day) and BMI (kg/m^2^).

### Ethical approval

Couples attending the fertility clinic at the UMCG are informed about possible use for research purposes of their anonymised medical data and spare serum samples that have been initially collected for diagnostic purposes, and a no-objection procedure is followed. Patients participating in the present study had not objected to their data and sera being used anonymously, and Institute Review Board approval was obtained from Amsterdam University Medical Centres (Letter reference: # 10.17.0046).

### TFI

TFI was based on laparoscopy findings exclusively, as laparoscopy is considered the reference standard for diagnosing adhesions and tubal patency. In this study, TFI was defined as extensive peri-adnexal adhesions and/or distal occlusion of at least one tube (Land *et al.*, 1998). Women with no abnormalities at laparoscopy were considered TFI-negative. Women who had few adhesions and/or proximal tubal occlusions were excluded, because they could not be grouped within our strict definition of TFI, but were also not considered completely TFI-negative.

### CAT

Serum samples were tested for the presence of *C. trachomatis* IgG antibodies with Medac ELISA plus (Medac GmbH, Wedel, Germany) in routine care during the fertility work-up. Tests were performed according to the manufacturer’s instructions. The cut-off for the Medac ELISA plus samples was 28 arbitrary units (AU)/ml, and samples with antibody levels ≥28 AU/ml were considered positive and <28 AU/ml negative.

### CRP testing

CRP was analysed in thawed sera using a high-sensitivity CRP ELISA kit outside of routine care in a research setting (Alpha Diagnostics, San Antonio, TX, USA, https://www.4adi.com/objects/catalog/product/extras/1000.pdf). Levels between 3.0 and 10.0 mg/L are considered to represent a chronic low-grade inflammation and were defined as positive in the present study, whereas results <3.0 and >10.0 mg/L were considered negative.

### Statistical analyses

Considering the small sample size, baseline characteristics were not statistically analysed for differences. Logistic regression analyses were performed for the association between seCRP levels and TFI in CAT-positive women. The findings were adjusted for the baseline characteristics BMI, endometriosis and smoking (Table I) by including them as covariates in the logistic regression analyses. For patients suffering from chronic diseases unrelated to TFI that can affect CRP levels (e.g. auto-immune diseases, allergies, asthma and psoriasis) (Windgassen *et al.*, 2011), separate analyses were performed. ORs and 95% CIs were calculated using IBM SPSS 22.0 (IBM Corp., Armonk, NY, USA).

**Table I:** Patient characteristics in 64 CAT-positive women who underwent laparoscopy for assessment of tubal factor infertility.

	**TFI negative (*n* = 41)**	**TFI positive (*n* = 23)**
Age (years), mean (±SD)	30.7 (4.8)	30.6 (5.4)
Infertility at intake (years), mean (±SD)	1.3 (1.4)	1.4 (1.1)
Slightly elevated CRP level^a^, *n* (%)	12 (29.3)	7 (30.4)
CRP level (mg/L), mean (±SD)	2.8 (3.4)	3.2 (3.5)
BMI (kg/m^2^), mean (±SD)	23.6 (3.8)	22.8 (2.6)
Smoking (cigarettes/day), mean (±SD)	3.1 (6.3)	3.0 (4.4)
Chronic disease^b^, *n* (%)	5 (11.6)	3 (13.0)
Endometriosis^c^, *n* (%)	3 (2.4)	3 (4.3)

CAT: *Chlamydia* IgG antibody test, TFI: tubal factor infertility, CRP: C-reactive protein

^a^CRP level 3.0–10.0 mg/L (indicating low-grade inflammation). ^b^Allergies, asthma, Crohn’s disease, eczema, hay fever, hypercholesterolemia and psoriasis. ^c^Peritoneal lesions with adhesions, endometrioma or deep infiltrating endometriosis

## Results

The initial cohort consisted of 887 patients, of whom 101 (11.4%) had a positive CAT result. After excluding patients who did not undergo laparoscopy or did not fulfil the strict definition of TFI after laparoscopy, of whom not enough spare serum for CRP testing was available or had missing data, 64 (7.2%) patients were analysed (Fig. 1). Baseline characteristics were summarised for both 41 TFI-negative and 23 TFI-positive patients (Table I). As shown in Table II, seCRP level was not associated with TFI and also not after adjusting for BMI, endometriosis and smoking. When analysed separately for patients who did not have a chronic disease that might cause elevated CRP levels (*n* = 56), the association remained non-significant in both the unadjusted analysis and the analyses adjusted for BMI, endometriosis and smoking (Table II).

**Table II:** Logistic regression results in CAT-positive women to analyse a potential correlation between slightly elevated CRP and TFI.

	**TFI (*n* = 64)** **Odds ratio (95% CI)**	**TFI excluding chronic diseases** ^**b**^ **(*n* = 56)** **Odds ratio (95% CI)**
seCRP^a^ *Unadjusted*	1.1 (0.4–3.2)	1.2 (0.4–3.9)
seCRP^a^ *Adjusted for BMI, endometriosis and smoking*	1.0 (0.3–3.3)	1.2 (0.4–4.0)

seCRP: slightly elevated CRP

^a^CRP-level 3.0–10.0 mg/L (indicating low-grade inflammation). ^b^Allergies, asthma, Crohn’s disease, eczema, hay fever, hypercholesterolemia and psoriasis

## Discussion

In our study population of infertile CAT-positive women, seCRP levels were not associated with TFI (adjusted OR 1.01; 95% CI 0.31–3.29). In this independent cohort of 64 women, we could not confirm the results of an earlier retrospective study by den Hartog *et al.* (2005), who showed an improvement of TFI prediction by adding a CRP test to CAT in 22 CAT-positive women. In both studies, the initial patient populations are comparable in terms of referral to Dutch fertility clinics and receiving comparable fertility work-up, but there are some differences between the two studies. Den Hartog *et al.* (2005) considered all patients without extensive peri-adnexal adhesions and/or distal occlusion of at least one tube as TFI-negative, while we only included women without any abnormalities as TFI-negative. In contrast to their analyses, we corrected for confounders from the literature, despite the small sample size, to illustrate the potential effect and value of these adjustments. Furthermore, the previous study used a different CRP test and cut-off value (1–10 mg/L). The cut-off used in the present study (3–10 mg/L) was based on recent publications on cardiovascular disease (Kushner and Antonelli, 2015), but nonetheless, when lowering the cut-off in our study to the levels used in the den Hartog study, the association between CRP and TFI remained non-significant (data not shown). Besides differences in the designs between these two studies, characteristics inherent to CRP as a marker for low-grade chronic inflammation could also have contributed to our non-confirmative results.

CRP is a general marker for inflammation. While we corrected for relevant confounders that were documented for the study population, i.e. BMI, endometriosis and smoking (Windgassen *et al*., 2011), other factors may have contributed to not finding an association between CRP and TFI. CRP was measured only once in our study population, and it was unknown on which day of the menstrual cycle the samples were taken. Since CRP is influenced by hormonal levels (Gaskins *et al*., 2012)—oestrogen lowers CRP, progesterone increases CRP levels—it is theoretically possible that this factor was not equally distributed in our study population. Furthermore, while we excluded known chronic disorders related to low-grade inflammation, undocumented disorders or nonspecific metabolic stress (Antonelli and Kushner, 2017) can also have been present in some patients. Moreover, while CRP is stimulated through pro-inflammatory cytokines that play a role in cell damage, fibrosis and scarring, these cytokines are also necessary to clear an infection (Menon *et al*., 2015). This might explain why that, while CRP is a marker for low-grade persisting inflammation, it may not be a valid marker for tissue damage and *C. trachomatis*-related TFI.

A limitation of our study is its retrospective design, small sample size and potential verification bias. Only CAT-positive women that underwent laparoscopy were included, leading to a small sample size. Although the initial cohort consisted of 887 women, only 101 (11.4%) were CAT-positive. A low prevalence of CAT-positivity in infertile women of between 6 and 16% has been reported by others as well (Keltz *et al*., 2013; Logan *et al*., 2003; Rantsi *et al*., 2018b). Laparoscopy is considered the reference test for diagnosing TFI, but not all CAT-positive women underwent this invasive procedure. Finally, women who had only a few adhesions or proximal tubal occlusions were excluded from the analyses as few adhesions were not considered to compromise fertility, and we had previously shown that CAT is not a suitable test to identify proximal tubal pathology (Land *et al*., 1998). Although we included consecutive patients in a large fertility centre for a period of 9 years, this resulted in a homogeneous cohort of 64 eligible women. Nonetheless, a strength of our study lies in the real-life situation where the patient population originated.

In cardiovascular disease patients, CRP has been shown to be a useful marker of persisting low-grade inflammation and the risk of late complications, and in a previous study in infertile women promising results were seen when combining CAT and seCRP for estimating the risk of TFI. In the present study in CAT-positive infertile women, we could not confirm these findings and found that CRP is not a suitable marker for identifying a subgroup at highest risk for TFI. As there is a clinical need for adequate non-invasive screening tests for TFI, to enable preselection of patients for subsequent invasive diagnostic testing by HSG or laparoscopy, several other inflammatory factors to predict the course of *C. trachomatis* infection have been studied, such as heat shock protein 60 and antibodies to specific chlamydial proteins (TroA and HtrA) (Hjelholt *et al*., 2011; Rantsi *et al.*, 2018a; Tiitinen *et al.*, 2006). A sensitive and specific serum marker has not been found so far. Another study approach to identify women at high risk for TFI could be making use of genetic markers of the host’s immune system, as 40% of the variation in the course of infection by *C. trachomatis* can be explained by host genetics (Bailey *et al*., 2009). Healthcare providers feel that they would perform such a genetic test if proven accurate, (cost-)effective and accompanied with professional training (Malogajski *et al.*, 2017). Clinical validity and utility remain to be established to evaluate if implementation of such testing for host genetic factors in the fertility work-up will improve the prediction of *C. trachomatis*-induced TFI.

## Authors’ roles

M.E.J., S.A.M. and J.A.L. were responsible for the design and oversight of this study. S.A.M. and S.O. obtained the funding. J.A.L., E.F.v.E. and M.L.G. reviewed the medical charts. S.O. and E.F.v.E. consulted on the study methods. All analyses, interpretation of data and drafting were conducted by M.E.J. with significant contribution from all authors. All authors approved the final manuscript.

## Funding

European EuroTrans-Bio Grant (110012 ETB); Eurostars grant (E!9372).

## Conflict of interest

S.A.M., a full-time employee of Amsterdam University Medical Centres location VUMC (0.56 fte) and the Maastricht University Medical Center (0.44 fte), is the founder (2011) and CEO of TubaScan Ltd, a spin-off company, Dept. of Medical Microbiology and Infection Prevention, Amsterdam UMC, Location VUmc, Amsterdam, the Netherlands. S.O. and E.F.v.E. at the time of conducting this research had a partial appointment at TubaScan Ltd.

## References

[ref1] Antonelli M, Kushner I It’s time to redefine inflammation. FASEB J 2017;31:1787–1791. 28179421 10.1096/fj.201601326R

[ref2] Bailey RL, Natividad-Sancho A, Fowler A, Peeling RW, Mabey DC, Whittle HC, Jepson AP Host genetic contribution to the cellular immune response to *Chlamydia trachomatis*: heritability estimate from a Gambian twin study. Drugs Today (Barc) 2009;45:45–50. 20011694

[ref3] Broeze KA, Opmeer BC, Coppus SF, van Geloven N, Alves MF, Ånestad G, Bhattacharya S, Allan J, Guerra-Infante MF, den Hartog JE et al. Chlamydia antibody testing and diagnosing tubal pathology in subfertile women: an individual patient data meta-analysis. Hum Reprod Update 2011;17:301–310. 21227996 10.1093/humupd/dmq060

[ref4] Budrys NM, Gong S, Rodgers AK, Wang J, Louden C, Shain R, Schenken RS, Zhong G *Chlamydia trachomatis* antigens recognized in women with tubal factor infertility, normal fertility, and acute infection. Obstet Gynecol 2012;119:1009–1016. 22525912 10.1097/AOG.0b013e3182519326 PMC4608258

[ref5] den Hartog JE, Land JA, Stassen FR, Kessels AG, Bruggeman CA Serological markers of persistent C. trachomatis infections in women with tubal factor subfertility. Hum Reprod 2005;20:986–990. 15640255 10.1093/humrep/deh710

[ref6] Gabay C, Kushner I Acute-phase proteins and other systemic responses to inflammation. NEJM 1999;340:448–454. 9971870 10.1056/NEJM199902113400607

[ref7] Gaskins AJ, Wilchesky M, Mumford SL, Whitcomb BW, Browne RW, Wactawski-Wende J, Perkins NJ, Schisterman EF Endogenous reproductive hormones and C-reactive protein across the menstrual cycle: the BioCycle Study. Am J Epidemiol 2012;175:423–431. 22306563 10.1093/aje/kwr343 PMC3282877

[ref8] Hjelholt A, Christiansen G, Johannesson TG, Ingerslev HJ, Birkelund S Tubal factor infertility is associated with antibodies against *Chlamydia trachomatis* heat shock protein 60 (HSP60) but not human HSP60. Hum Reprod 2011;26:2069–2076. 21642639 10.1093/humrep/der167

[ref9] Johnston SC, Messina LM, Browner WS, Lawton MT, Morris C, Dean D C-reactive protein levels and viable *Chlamydia pneumoniae* in carotid artery atherosclerosis. Stroke 2001;32:2748–2752. 11739967 10.1161/hs1201.099631

[ref10] Keltz MD, Sauerbrun-Cutler MT, Durante MS, Moshier E, Stein DE, Gonzales E Positive *Chlamydia trachomatis* serology result in women seeking care for infertility is a negative prognosticator for intrauterine pregnancy. Sex Transm Infect 2013;40:842–845. 10.1097/OLQ.0000000000000035 24113404

[ref11] Kushner I, Antonelli MJ What should we regard as an ‘elevated’ C-reactive protein level? Ann Intern Med 2015;163:326. 26280429 10.7326/L15-5126

[ref12] Land JA, Evers JL, Goossens VJ How to use Chlamydia antibody testing in subfertility patients. Hum Reprod 1998;13:1094–1098. 9619578 10.1093/humrep/13.4.1094

[ref13] Land JA, Gijsen AP, Kessels AG, Slobbe ME, Bruggeman CA Performance of five serological chlamydia antibody tests in subfertile women. Hum Reprod 2003;18:2621–2627. 14645182 10.1093/humrep/deg479

[ref14] Logan S, Gazvani R, McKenzie H, Templeton A, Bhattacharya S Can history, ultrasound, or ELISA chlamydial antibodies, alone or in combination, predict tubal factor inferility in subfertile women? Hum Reprod 2003;18:2350–2356. 14585886 10.1093/humrep/deg471

[ref15] Malogajski J, Jansen ME, Ouburg S, Ambrosino E, Terwee CB, Morré SA The attitudes of Dutch fertility specialists towards the addition of genetic testing in screening of tubal factor infertility. Sex Reprod Healthc 2017;12:123–127. 28477924 10.1016/j.srhc.2017.04.001

[ref16] Menon S, Timms P, Allan JA, Alexander K, Rombauts L, Horner P, Keltz M, Hocking J, Huston W Human and pathogen factors associated with *Chlamydia trachomatis*-related infertility in women. Clin Microbiol Rev 2015;28:969–985. 26310245 10.1128/CMR.00035-15 PMC4548260

[ref17] Pearson TA, Mensah GA, Alexander RW, Anderson JL, Cannon RO, Criqui M, Fadl YY, Fortmann SP, Hong Y, Myers GL et al. Markers of inflammation and cardiovascular disease. Application to clinical and public health practice. A statement for healthcare professionals from the Centers for Disease Control and Prevention and the American Heart Association. Circulation 2003;107:499–511. 12551878 10.1161/01.cir.0000052939.59093.45

[ref18] Rantsi T, Joki-Korpela P, Hokynar K, Kalliala I, Öhman H, Surcel H, Paavonen J, Tiitinen A, Puolakkainen M Serum antibody response to *Chlamydia trachomatis* TroA and HtrA in women with tubal factor infertility. Eur J Clin Microbiol Infect Dis2018a;37:1499–1502. 29777489 10.1007/s10096-018-3276-9

[ref19] Rantsi T, Öhman H, Puolakkainen M, Bloigu A, Paavonen J, Surcel HM, Tiitinen A, Joki-Korpela P Predicting tubal factor infertility by using markers of humoral and cell-mediated immune response against *Chlamydia trachomatis*. Am J Reprod Immunol2018b;80:e13051. 30281189 10.1111/aji.13051

[ref20] Tiitinen A, Surcel H-M, Halttunen M, Birkelund S, Bloigu A, Christiansen G, Koskela P, Morrison SG, Morrison RP, Paavonen J *Chlamydia trachomatis* and chlamydial heat shock protein 60-specific antibody and cell-mediated responses predict tubal factor infertility. Hum Reprod 2006;21:1533–1538. 16478761 10.1093/humrep/del014

[ref21] Windgassen EB, Funtowicz L, Lunsford TN, Harris LA, Mulvagh SL C-reactive protein and high-sensitivity C-reactive protein: an update for clinicians. Postgrad Med 2011;123:114–119. 10.3810/pgm.2011.01.2252 21293091

